# Fabrication of Nanopore in MoS_2_-Graphene vdW Heterostructure by Ion Beam Irradiation and the Mechanical Performance

**DOI:** 10.3390/nano12020196

**Published:** 2022-01-07

**Authors:** Xin Wu, Ruxue Yang, Xiyue Chen, Wei Liu

**Affiliations:** School of Chemical Engineering and Technology, Sun Yat-sen University, Zhuhai 519082, China; yangrx7@mail2.sysu.edu.cn (R.Y.); chenxy795@mail2.sysu.edu.cn (X.C.)

**Keywords:** MoS_2_-graphene vdW heterostructure, nanopore fabrication, ion beam irradiation, mechanical performance

## Abstract

Nanopore structure presents great application potential especially in the area of biosensing. The two-dimensional (2D) vdW heterostructure nanopore shows unique features, while research around its fabrication is very limited. This paper proposes for the first time the use of ion beam irradiation for creating nanopore structure in 2D vdW graphene-MoS_2_ heterostructures. The formation process of the heterostructure nanopore is discussed first. Then, the influence of ion irradiation parameters (ion energy and ion dose) is illustrated, based on which the optimal irradiation parameters are derived. In particular, the effect of stacking order of the heterostructure 2D layers on the induced phenomena and optimal parameters are taken into consideration. Finally, uniaxial tensile tests are conducted by taking the effect of irradiation parameters, nanopore size and stacking order into account to demonstrate the mechanical performance of the heterostructure for use under a loading condition. The results would be meaningful for expanding the applications of heterostructure nanopore structure, and can arouse more research interest in this area.

## 1. Introduction

Nanopore technology refers to the creation and applications of nanometer pores in membrane structures. Since the size of nanopore channels is comparable to that of typical biomolecules, it is possible to use nanopores for detecting various biomolecules. The first demonstration of nanopores in biosensing was achieved in 1996 [1]. Thereafter, many research efforts have been made to uncover the underlying mechanisms of nanopore-based biosensors and to improve the efficiency and sensitivity of detection [2,3,4,5]. Nanopore-based single molecule sequencing was even taken as one of the most promising techniques to realize the objective of the “$1000 genome” project [6]. Currently, there are four major types of nanopores: biological nanopores, solid-state nanopores, two dimensional (2D) nanopores, and hybrid nanopores. Among them, the 2D nanopores (nanopore creation in 2D materials) stand out due to the unique features of the 2D materials [7,8]. For example, the subnanometer thickness of the 2D nanopore is close to the spatial interval of neighboring nucleic acid bases, giving the ability to achieve gene sequencing at a single-base level. The high carrier mobility of 2D materials also enables the detection of biomolecules with great sensitivity. Besides the applications in biosensing, 2D nanopore technology has also been widely used in ion filtration, sea water desalination, gas separation, etc. [9,10,11]. 

2D nanopore materials can be fabricated by many techniques [12,13,14], which can be categorized into “top-down” methods, i.e., directly creating the pore structure in membrane materials, and “bottom-up” methods, i.e., synthesizing nanopores during the growth of 2D materials. Recently, Su et al. [15] comprehensively reviewed the approaches applied in nanopore fabrication in 2D materials, and they divided the fabrication methods into four types: energetic particle impact, chemical reaction, physical and chemical methods, and others. Most of the fabrication methods aim at obtaining nanopore arrays with high pore density, which are usually applied in water desalination [9], gas separation [11], and energy conversion [16]. In contrast, some applications such as biosensors require that a single pore is created in the membrane. For this scenario, the methods of electrical pulses [17], transmission electron microscope sculpting [18], and ion beam irradiation [19] are more suitable. Due to the flexibility in adjusting the irradiation parameters and the ability to create pore structures with diameters from subnanometer to tens of nanometers, ion beam irradiation is recognized as the most efficient technique in fabricating nanopores for biosensing. Consequently, many theoretical and experimental studies have been conducted to illustrate the general phenomena and underlying mechanisms of the ion beam fabrication of 2D nanopore structures [20,21,22]. 

The 2D materials adopted for the creation of nanopores mainly include graphene, MoS_2_ and h-BN [23,24]. For applications of 2D nanopores in biosensing, a large electric field is often applied to drive the biomolecules through the nanopore, which results in rapid penetration, limiting the resolution of detection. Moreover, this method only works for biomolecules with net charges. Therefore, it is important to find suitable charge-independent methods for nanopore biosensing. Recently, the nanopores in 2D vdW heterostructures, which are synthesized by vertically stacking two different 2D crystals [25], were demonstrated to be able to drive the penetration of biomolecules by the difference in binding affinities for each 2D surface, rather than the external electrical field [26,27,28]. The 2D vdW heterostructure nanopore could slow down the translocation speed of the biomolecules as well, which is significant for applications such as DNA sequencing. Therefore, the applications of 2D vdW heterostructure nanopores in biosensing present more advantages. There is, however, limited research around the synthesis of nanopores in vdW heterostructures, especially for the fabrication of nanopores in 2D vdW heterostructures by ion beam irradiation.

Hence, this paper focuses on the creation of nanopore structure in 2D vdW heterostructures using Ar ion beam irradiation, for which classical molecular dynamics (MD) simulation method is adopted, while graphene/MoS_2_ (G/M, graphene layer facing the ion beam) heterostructure is taken as the representative structure due to its well-demonstrated performance in biosensing [26,28]. The nanopore formation process and the influence of the irradiation parameters are revealed. By switching the stacking order of the 2D crystals, i.e., generating the MoS_2_/graphene (M/G) heterostructure, distinct phenomena can be observed. The mechanical behavior of the heterostructure and nanopore under tensile strain is investigated for actual applications. 

## 2. Simulation Models

**Figure 1:**
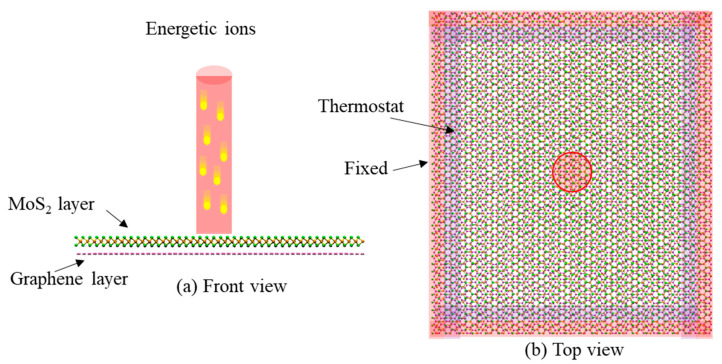
Simulation models of the M/G nanopore fabrication by ion beam irradiation. (**a**) Front view, and (**b**) top view.

In this study, the classical MD simulation method is applied through the large scale atomic/molecular massively parallel simulator (LAMMPS) software [29]. LAMMPS is an open source package with a record of successful demonstrations in describing the interactions between ions and 2D materials [30,31,32]. Ar ions are selected for the irradiation, since they are rich as an ion source in experiments [33,34] and widely used in other simulation studies [22]. Monolayer graphene-MoS_2_ (G/M or M/G) heterostructures are the impact target. The nanopore in a heterostructure is generated through the transfer of momentum energy from energetic Ar to the heterostructure layers. To mimic this process, a hybrid atomic potential is adopted. The interaction between Ar ions and atoms in a heterostructure is described by the Ziegler–Biersack–Littmark (ZBL) universal repulsive potential [35], which can present atomic interaction at small separation during the irradiation. The interaction between the atoms in MoS_2_ is captured by a second-generation reactive empirical bond-order (REBO) potential [36], and the interaction between the atoms in graphene is considered as an adaptive intermolecular reactive bond order (AIREBO) potential [37]. The vdW interaction between MoS_2_ and graphene crystal is modeled by the Lennard–Jones (LJ) potential [38], which is parameterized according to [39,40]. The simulation model is illustrated in Figure 1, which presents an in-plane size of 115 × 130 Å with 11,124 atoms. The in-plane directions of the simulation box are assigned with periodic boundary conditions, while the out-of-plane direction is fixed to allow the injection of ion beams. During the ion irradiation process, the outermost several layers of the heterostructure are fixed, and several adjacent layers are assigned with Berendsen temperature control [41], as shown in Figure 1b. This strategy could prevent the heterostructure from overall movement, and minimize the side effects of the ion induced pressure wave as well. The system is first equilibrized through an NVT ensemble at 300 K for enough time to reach a relaxed state. Then, consecutive Ar ions are randomly emitted from a cylinder with the axis located at the center of the heterostructure. The radius of the irradiated spot is 10 Å which can be achieved by using a focused ion beam technique in the experiment [42], and the cylinder is 40 Å on top of the heterostructure plane. Ar ions are irradiated every 1000 timesteps with a step size of 0.5 fs to achieve a certain dose, during which process the system is stabilized at 300 K. In this study, various ion doses from 1.59 × 10^15^ /cm^2^ to 2.54 × 10^16^ /cm^2^ and various ion energies from 40 eV to 5000 eV are considered to uncover the influence of ion parameters on the fabrication of the nanopore. The studied ranges of ion dose and ion energy also match the experimental conditions well [33,34]. After the irradiation stage, the irradiated structures are annealed at 2000 K for enough time to mimic the long-time annealing process in actual experiments. Finally, the system is cooled to room temperature, and kept at this state. The above simulation scheme can reveal the phenomena during the ion irradiation process, as demonstrated by many researchers [32,43].

After the ion irradiation and annealing process, the obtained atomic coordinates of the heterostructures with nanopores are extracted and a uniaxial tensile stretching process is applied to investigate the mechanical performance of the nanopore structure. One side of the irradiated structure is fixed and the other side is stretched with a strain rate of 0.0005 ps^−1^. The stress information for each atom is first calculated and then stress along the stretching direction is summed up for all the atoms to get the overall stress of the whole system. The overall stress values are averaged every 500 timesteps for outputting to reduce the fluctuation of the data. Thereafter, the stress-strain relationship for each system is obtained and plotted. To show the dynamics of stress evolution for each atom during the stretching process, the per-atom stress information is also outputted, and the Open Visualization Tool (OVITO) software [44] is adopted for visualizing. The other configurations are visualized by the Visual Molecular Dynamics (VMD) program [45].

## 3. Results and Discussion

### 3.1. Dynamic Formation Process of Nanopore

During ion irradiation, the irradiated particle would interact with atoms in the 2D crystals, which may lead to four phenomena, i.e., reflection, absorption, embedment, or penetration, depending on the impact energy [46]. The momentum energy would be transferred from the Ar ions to the atoms in 2D vdW heterostructure, which induces the fluctuation of the heterostructure plane and generates knocked-out atoms if the irradiation energy is high enough.

**Figure 2:**
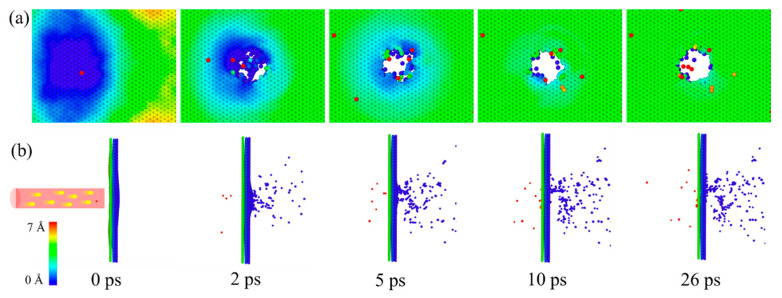
Structural evolution of the G/M heterostructure under ion irradiation. (**a**) Front view, and (**b**) left view. Ion energy is 200 eV, ion dose is 1.27 × 10^16^ /cm^2^. The atoms are colored according to the z coordinates. z is defined as the coordinate axis along the irradiation direction.

Figure 2 shows the dynamics of formation of the nanopore structure in G/M heterostructure with an irradiation parameter pair of 200 eV, 1.27 × 10^16^ /cm^2^. The colors are used to mark the coordinates of each atom, so that we can obtain the displacement of the atoms under different irradiation times. The initial blue color indicates the random fluctuation of the structure during the annealing process (time = 0 ps). After being irradiated, the atoms at the irradiated area would move along the irradiation direction and separate from the heterostructure plane at a certain energy level, generating irregular defects when the irradiation time is short (time = 2 ps). The damaged area is initially characterized rough edges and dangling molecular chains. With the increase of irradiation time, more atoms in the heterostructure would be sputtered and the nanopore structure would be initiated and grown radially (time = 5 ps). The dangling molecular chains would be gradually cleared up, resulting in a smoother pore structure (time = 10 ps). Eventually, the nanopore becomes stabilized, and the atoms at the pore edges fluctuate only slightly because there is no more impact from the irradiation (time = 26 ps). Figure 2b gives the left views of the structure during the irradiation process, which reveals a large displacement for the atoms at the pore edge at the beginning, while there is a small displacement for the edge atoms when the pore structure gets formed. The random location of the red colored atoms indicates that there are some rebounded atoms during the generation of the nanopore. 

### 3.2. Influence of Ion Irradiation Energy and Dose

**Figure 3:**
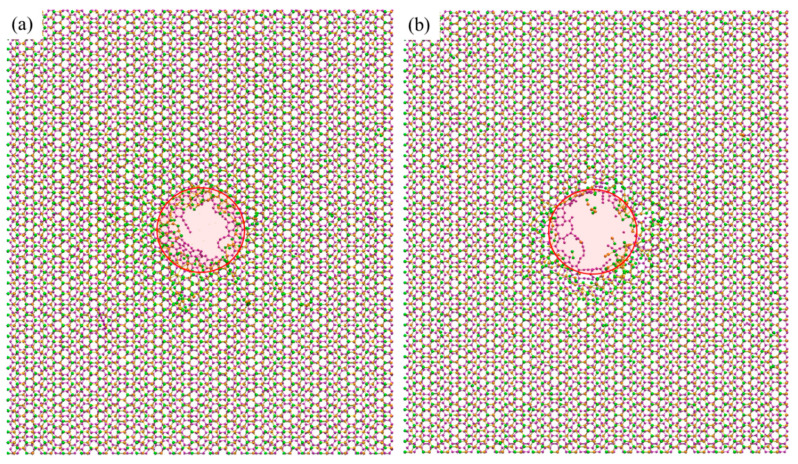
Morphologies of the G/M heterostructure under ion irradiation with a parameter pair of (**a**) 80 eV, and 2.54 × 10^16^ /cm^2^. (**b**) 5000 eV, 3.18 × 10^15^ /cm^2^. The red colored circle is used to indicate the irradiation area.

As illustrated in the above, ion irradiation may be able to create a nanopore in the heterostructure at a certain ion irradiation energy and dose. However, this does not mean that the desired pore structure can be formed at any ion parameter. As shown in Figure 3, when the ion energy is small (80 eV), the formed pore is far from the designed structure (red circled area), regardless of the ion irradiation dose (2.54 × 10^16^/cm^2^ is actually a relatively high ion dose). Meanwhile, if the ion dose is small (3.18 × 10^15^ /cm^2^), the generated pore would also be far from the designed form, regardless of the ion irradiation energy (5000 eV is actually a relatively high ion energy). Therefore, to achieve a nanopore with desired structure, it is necessary to carefully control the ion irradiation parameters. 

**Figure 4:**
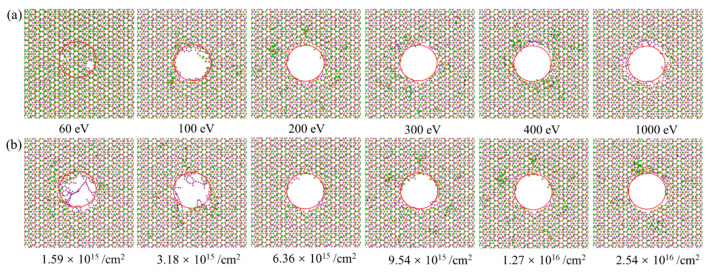
Configurations of the G/M heterostructure under ion irradiation with different ion energies and ion doses. (**a**) The influence of ion energy under a constant ion dose of 1.27 × 10^16^ /cm^2^. (**b**) The influence of ion dose under a constant ion energy of 200 eV. The red colored circle is used to indicate the irradiation area.

Figure 4 gives the morphologies of the G/M heterostructure irradiated by ions with energies from 60 eV to 1000 eV, and doses from 1.59 × 10^15^ /cm^2^ to 2.54 × 10^16^ /cm^2^. As shown in Figure 4a, when the irradiation energy is 60 eV, the irradiated area presents a morphology more like irregular defects, rather than a pore structure. The area gradually develops into a nanopore structure when the ion energy increases from 100 eV to 200 eV. It is seen that under ion irradiation energy above 200 eV (i.e., from 200 eV to 1000 eV), there is little change for the pore structure. As depicted in Figure 4b, with an ion irradiation dose of 1.59 × 10^15^ /cm^2^ and 3.18 × 10^15^ /cm^2^, there would be obvious long residual carbon bonds in the graphene plane, while the residual bonds in the MoS_2_ layer are very limited, indicating an easier knock-out phenomenon in MoS_2_ when compared to graphene. Under higher irradiation dosage, these long residual carbon bonds would be largely cleared up and a nanopore with high quality would be formed. The residual chains would be largely reduced, though they still exist around the nanopore edge. These residual bonds are usually chemically active, providing ideal sites for the hydrogenation [9] and nitridation [10] of the nanopore structure, which is important for some application scenarios. Even though ion irradiation with a higher energy and larger dose could be helpful to achieve an ideal nanopore, the redundant energy and dose may be detrimental to the experimental equipment, which means that the derivation of an optimized irradiation parameter pair (the least irradiation energy and dose for generating a good-quality pore structure) is needed.

**Figure 5:**
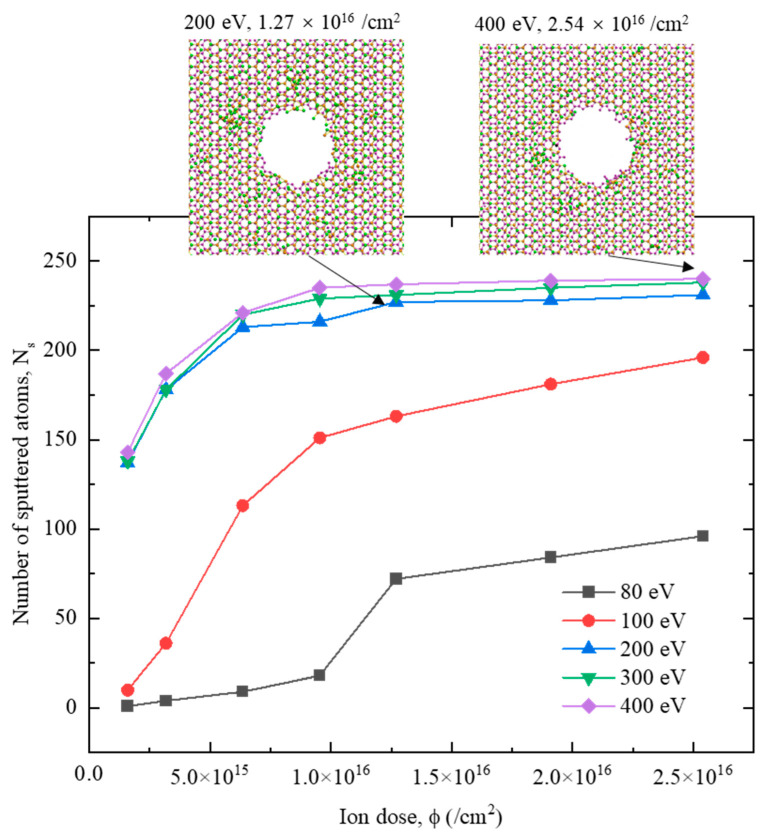
Number of sputtered atoms of the G/M heterostructure under ion irradiation with different ion energies and ion doses. The inserted figures show the configurations of 200 eV, 1.27 × 10^16^ /cm^2^, and 400 eV, 2.54 × 10^16^ /cm^2^, respectively.

Figure 5 plots the dependence of the number of sputtered atoms of the G/M heterostructure on ion irradiation energy and ion dose. It clearly shows that with the increase of ion irradiation energy and dose, the number of sputtered atoms for all the cases would increase. When the ion irradiation energy is low (80 eV and 100 eV), the number of sputtered atoms is small and increases quickly with the addition of the irradiation dose. It can be expected that under these two cases, the number of sputtered atoms would be further increased if the ion dose is raised beyond the studied range (2.54 × 10^16^ /cm^2^). When the irradiation energy is high (200 eV, 300 eV and 400 eV), the number of sputtered atoms is relatively large and a steady status is quickly achieved with the increase of ion irradiation dose. Moreover, the three cases, i.e., 200 eV, 300 eV and 400 eV, present very close data, indicating that 200 eV irradiation energy is large enough to generate the desired pore structure. Further increase of the irradiation energy has limited benefit to the quality of the nanopore, while it would introduce large side effects to the equipment in the experiment. Therefore, 200 eV is determined as the optimal ion irradiation energy. As for the influence of the ion irradiation dose, the number of sputtered atoms becomes stable after reaching the ion dose of 1.27 × 10^16^ /cm^2^, indicating 1.27 × 10^16^ /cm^2^ as the optimal ion irradiation dose. Therefore, the optimal parameter pair to obtain a high-quality nanopore is derived as 200 eV and 1.27 × 10^16^ /cm^2^. The detailed nanopore structures generated under ion irradiation of 200 eV, 1.27 × 10^16^ /cm^2^ and 400 eV, 2.54 × 10^16^ /cm^2^ are also compared in Figure 5, which shows that the optimal parameter pair is able to generate the desired structure, while increasing ion energy and dose has limited effect on the improvement of the nanopore quality.

### 3.3. Influence of the Stacking Order

**Figure 6:**
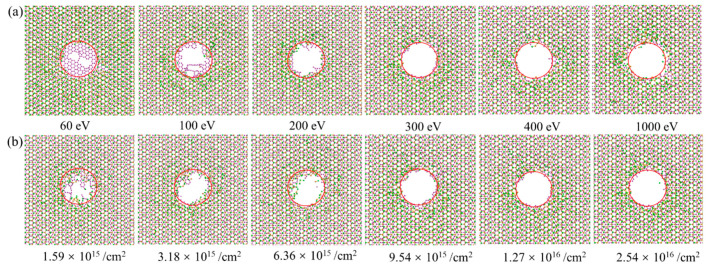
Configurations of the M/G heterostructure under ion irradiation with different ion energies and ion doses. (**a**) The influence of ion energy under a constant ion dose of 1.27 × 10^16^ /cm^2^. (**b**) The influence of ion dose under a constant ion energy of 300 eV. The red colored circle is used to indicate the irradiation area.

Graphene and MoS_2_ have different damage thresholds under ion beam irradiation. Therefore, there might be distinguishing irradiation phenomena if we switch the stacking order of the two layers. Figure 6 shows the configuration of M/G heterostructure (MoS_2_ facing the ion beam) under ion irradiation. It indicates that under a low irradiation energy (60 eV), the top MoS_2_ layer is quickly removed in the irradiated area, while the bottom graphene layer is largely retained with some dangling bonds generated. With the increase of irradiation energy, the graphene layer with the dangling bonds would be gradually cleared up, generating the MoS_2_/graphene nanopore structure (200 eV). The final configuration would be stable if the ion energy is large enough (above 300 eV). With the constant ion energy of 300 eV (Figure 6b), the low ion dose (1.59 × 10^15^/cm^2^) would result in irregular damage with a lot of dangling molecular chains in the irradiation area, while an increase of ion dose can lead to the final formation of the desired nanopore structure. 

**Figure 7:**
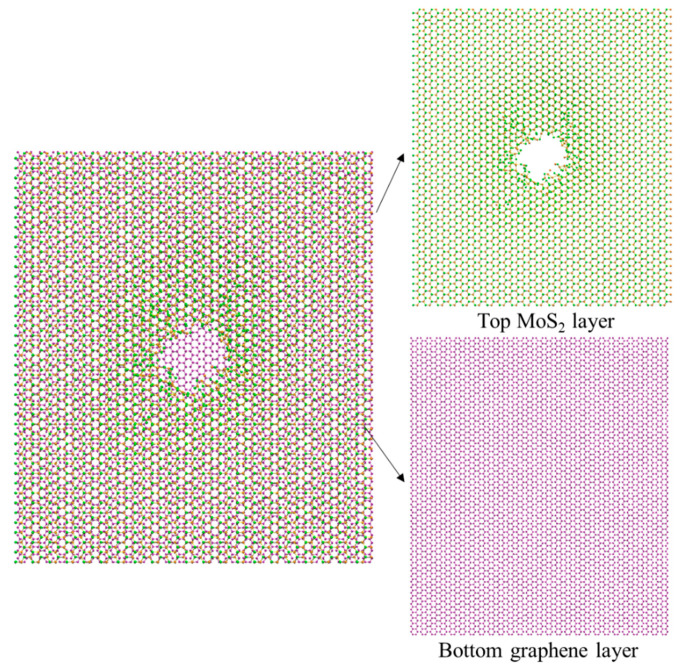
Configurations of the M/G heterostructure under ion irradiation with a parameter pair of 80 eV, 3.18 × 10^15^ /cm^2^.

Compared to the results in Figure 5, the configurations of the M/G heterostructure in Figure 6 demonstrate a similar phenomenon—that a nanopore structure in both heterostructures would be gradually generated with the increase of ion irradiation energy and dose, while it seems that the M/G heterostructure requires a higher irradiation energy for nanopore formation. The MoS_2_ and graphene layer in the M/G heterostructure gets damaged with an obvious order of precedence, i.e., the MoS_2_ gets damaged first while the damage to the graphene layer depends clearly on the irradiation energy. Therefore, we can suspect that by carefully controlling the ion irradiation energy and dose, a nanopore might be generated in the MoS_2_ layer only, while the graphene layer stays undamaged. Figure 7 shows that under the ion parameter pair of 80 eV, 3.18 × 10^15^ /cm^2^, the nanopore structure can be formed in the top MoS_2_ layer, with no damage in bottom graphene layer, which cannot be achieved in the case of G/M heterostructure. The selective creation of a nanopore in a MoS_2_ layer may have important applications which need experimental validation.

**Figure 8:**
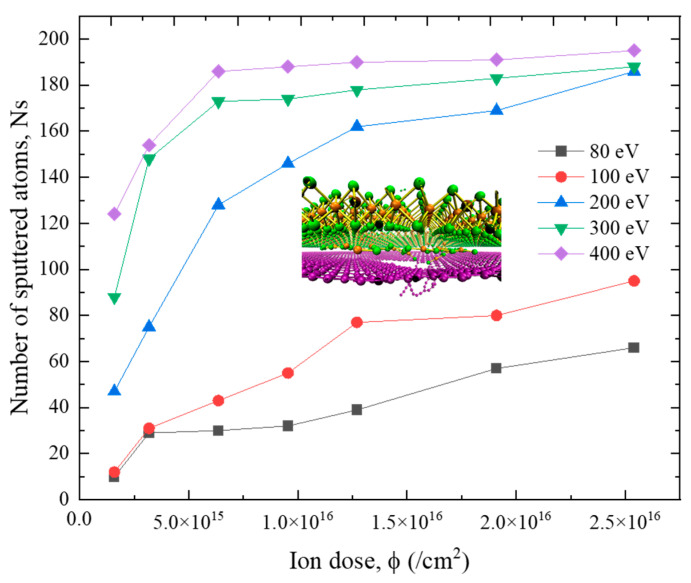
Number of the sputtered atoms of the M/G heterostructure under ion irradiation with different ion energies and ion doses. The inserted figure shows the cross-section enlarged view of the irradiated heterostructure.

To derive the optimal ion irradiation parameters for M/G heterostructure, the dependence of the number of sputtered atoms on ion irradiation energies and ion doses is plotted in Figure 8, from which it can be seen that the number of sputtered atoms would increase with the increase of ion irradiation energy and ion irradiation dose. For the cases of 80 eV, 100 eV and 200 eV, the number of sputtered atoms continuously increases within the studied ion dose range. However, for the cases of 300 eV and 400 eV, the number of sputtered atoms rises quickly at first and then reaches an equilibrium value at a dose of around 1.27 × 10^16^ /cm^2^. Combined with the results in Figure 6, we can determine that the optimal ion parameter pair for M/G heterostructure to achieve a good-quality nanopore is 300 eV, 1.27 × 10^16^ /cm^2^, under which condition the influence of redundant ions on the equipment is minimized. 

In addition, it is observed that for most of the studied cases, the number of sputtered atoms for M/G heterostructure is noticeably smaller than that for G/M heterostructure. This is because the graphene layer is more resistant to the ion irradiation when compared to the MoS_2_ layer [31,43]. For the G/M heterostructure, if the ion energy is high enough to knock out the atoms in graphene, then it is highly probable that the irradiated ions and sputtered atoms can also sputter the atoms in MoS_2_. However, for the M/G heterostructure, even though the irradiated ions can knock out the atoms in the MoS_2_ layer more easily, the residual energy of the irradiated ions and the sputtered Mo, S atoms may not be able to damage the graphene, generating the embedded atoms in the interlayer space, as shown in the inserted figure in Figure 8. The easily embedded atoms also lower the efficiency for nanopore creation in the M/G heterostructure, which means that a higher irradiation energy is required for nanopore formation in the M/G case.

### 3.4. Mechanical Properties of the As-Obtained Heterostructure Nanopore

**Figure 9:**
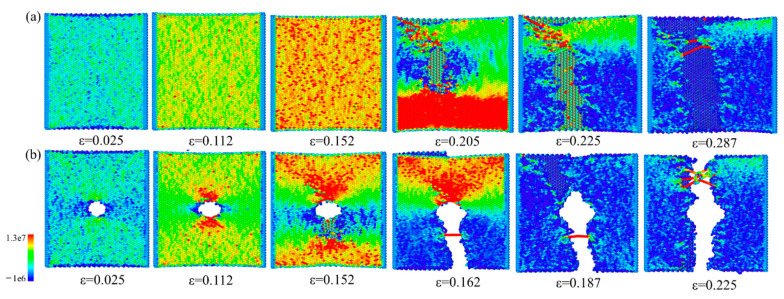
Snapshots of the G/M heterostructure morphology at different tensile strains during uniaxial stretching process. (**a**) Pristine heterostructure. (**b**) Heterostructure nanopore generated with an irradiation parameter pair of 200 eV, 1.27 × 10^16^ /cm^2^. The structures are colored according to the per-atom stress information along the horizontal direction, and the value has a unit of stress/(per-atom volume).

In actual applications, the nanopore is sometimes working under loading conditions [9,47]. Thus, the mechanical properties of the fabricated nanopore should be investigated. We conducted a uniaxial stretching process for the original and ion irradiated G/M heterostructures, and the results are shown in Figure 9, for which the configurations are colored according to the per-atom stress information along the stretching direction. It is seen that for the original heterostructure, the stress value for all the atoms would rise with the increase of tensile strain, and the structure would fracture at a random location when the stress is large enough. For the case of a heterostructure with a nanopore, the stress quickly concentrates around the nanopore structure under the stretching condition. Increase of the concentrated stress leads to the fracture of the heterostructure around the nanopore. Due to the stress concentration, the nanopore heterostructure gets fractured at a much smaller strain when compared to the original heterostructure (ε = 0.152 vs. ε = 0.205). For both cases, the fracture of structure starts from the top graphene layer, and the stress value is quickly diminished with the expansion of the cracks.

**Figure 10:**
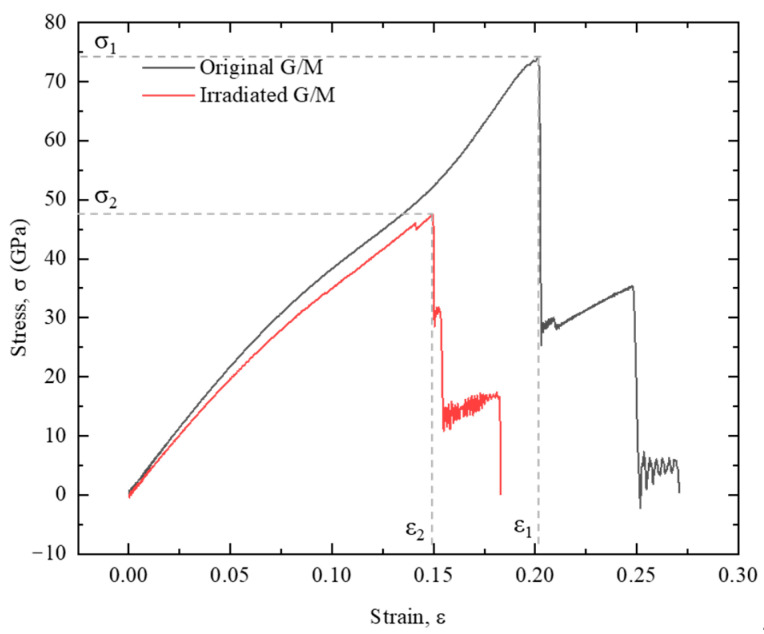
Stress-strain relationship of the G/M heterostructure with and without the ion irradiation process. The irradiation parameters are 200 eV, 1.27 × 10^16^ /cm^2^.

The stress-strain relationships of the heterostructures with and without nanopore are plotted in Figure 10. This reveals a multi-stage fracture process for both the original heterostructure and the heterostructure with a nanopore. As indicated in Figure 9, the fracture of the structure starts from the top graphene layer, and then the crack would start to initiate in the bottom MoS_2_ with the increase of tensile strain. Due to the existence of nanopore structure, the primary tensile fracture strain and stress at the first fracture stage are much smaller for the ion irradiated heterostructure (0.149, 47.2 GPa vs. 0.202, 73.8 GPa). 

**Figure 11:**
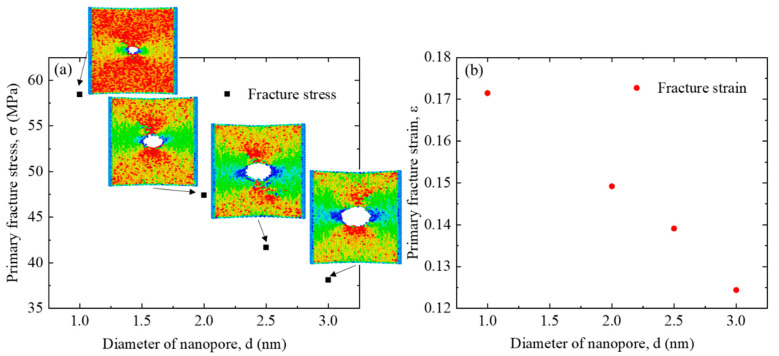
Influence of the G/M heterostructure nanopore size on the mechanical properties. (**a**) Primary fracture stress, and (**b**) primary fracture strain. The inserted figures show the per-atom stress distribution just before the fracture of the first stage.

Figure 11 depicts the influence of the as-fabricated nanopore size on the mechanical performance of the heterostructure. The ions have an irradiation parameter pair of 200 eV, 1.27 × 10^16^ /cm^2^. Figure 11 shows that both the primary fracture stress and fracture strain would reduce almost linearly with the increase of nanopore size. This is because the larger size nanopore would result in a higher stress concentration factor for 2D materials [48]. The inserted figures show the stress distribution of the structure just before the fracture happens. They clearly indicate that for the nanopore structure with a smaller size, a larger overall stress value is required to initiate the fracture, indicating a larger strain needed at the fracture point.

**Figure 12:**
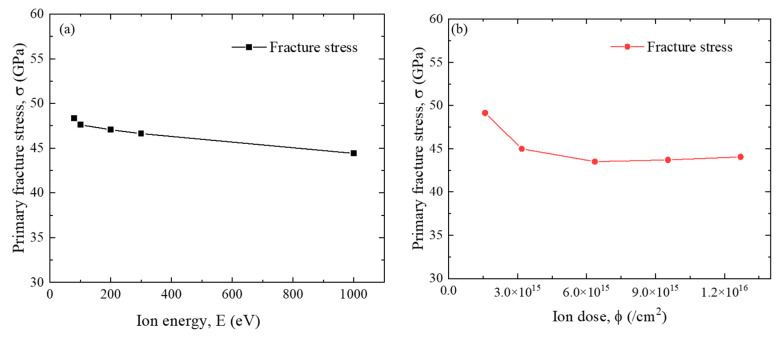
Influence of the (**a**) irradiation energy and (**b**) ion dose on the primary mechanical strength of G/M heterostructure. The ion dose for (**a**) is 1.27 × 10^16^ /cm^2^ and the ion energy for (**b**) is 200 eV, respectively.

Figure 12 shows the influence of ion energy and dose on the mechanical strength of the M/G heterostructure. Both the increase of ion energy and ion dose would result in an overall slight reduction of the primary fracture strength of the irradiated structure. This indicates that even though the low ion energy and ion dose only result in irregular damage in the structure, the reduction in mechanical strength is similar to that of the structure with a nanopore. Therefore, the influence of structural damage on the mechanical behavior of 2D heterostructures needs more attention.

**Figure 13:**
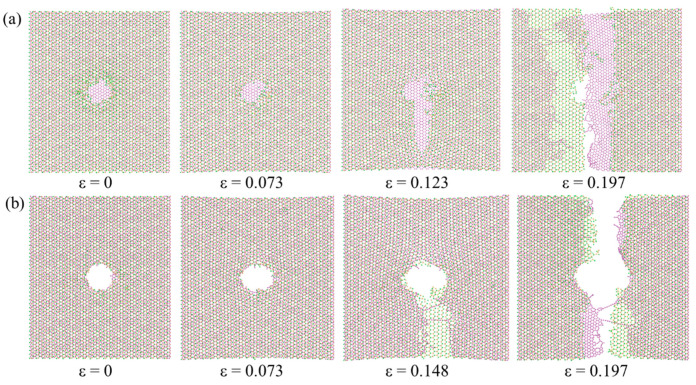
Dynamics of structural evolution of the M/G heterostructure nanopore under ion irradiation with irradiation parameters of (**a**) 80 eV, 3.18 × 10^15^ /cm^2^ and (**b**) 400 eV, 1.27 × 10^16^ /cm^2^.

Figure 13 shows the dynamics of structural evolution of the M/G heterostructure nanopore under ion irradiation with different irradiation parameters. It is seen that under the parameter pair of 80 eV, 3.18 × 10^15^ /cm^2^, there is nanopore formation only in the top MoS_2_ layer, while the bottom graphene layer is undamaged, resulting in the initiation of the crack in MoS_2_ layer, different from the case in Figure 9. In contrast, if we use the ion parameter pair of 400 eV, 1.27 × 10^16^ /cm^2^, a good-quality nanopore is formed in both the MoS_2_ and graphene layers, because of which the initiation of the crack happens in graphene layer again, similar to the case of G/M heterostructure. The phenomenon demonstrates that the damage sequence of the heterostructure nanopore can be controlled by adjusting the irradiation parameters. 

**Figure 14:**
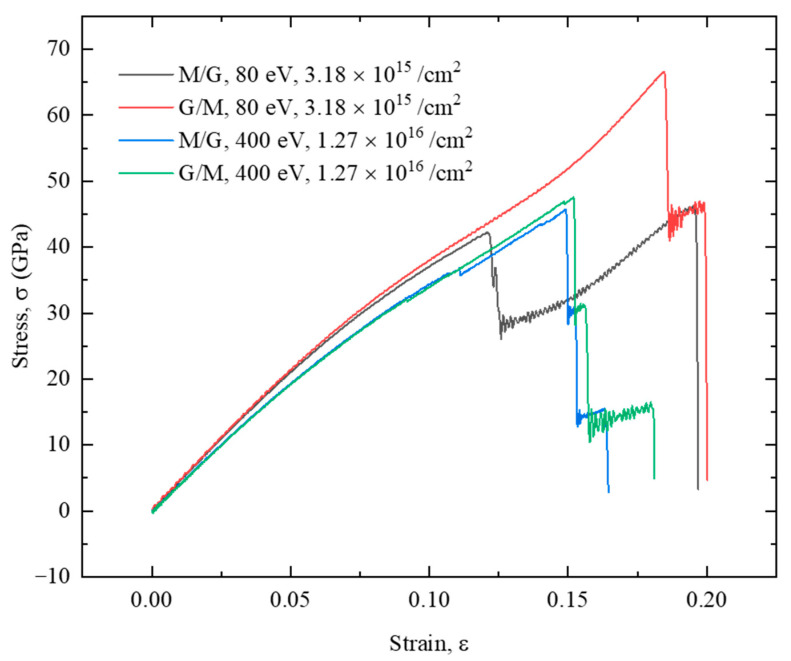
Stress-strain relationship of the G/M and M/G heterostructure with nanopore structure created by ion irradiation under different irradiation parameters.

Figure 14 plots the stress-strain relationship of the G/M and M/G heterostructures with the nanopore structure created by ion irradiation with the same parameter pair. It shows that under the irradiation with a parameter pair of 80 eV, 3.18 × 10^15^ /cm^2^, the MoS_2_ layer in the M/G heterostructure is severely damaged, generating a much reduced strength value for the first fracture stage. However, the graphene layer is well retained, leading to an increased fracture strength for the second fracture stage. For the G/M heterostructure, there is very limited damage on the heterostructure due to the shielding effect of the graphene structure. Thus, a much higher fracture strength is observed. When the structure is irradiated with a parameter pair of 400 eV, 1.27 × 10^16^ /cm^2^, both G/M and M/G heterostructures show similar mechanical properties because of the similar structural damage induced by the ion irradiation.

## 4. Conclusions

This paper studies the fabrication of nanopores in 2D vdW heterostructures by ion beam irradiation. The formation process of the nanopore structure, influencing factors and the uniaxial tensile properties are investigated. The main conclusions are as follows.

(1) The nanopore is fabricated by the sputtering of the atoms during ion irradiation. The process is characterized by initial formation of irregular defects. A nanopore with rough edges and dangling molecular chains is then generated, followed by the formation of a good-quality nanopore in the heterostructure.
(2) The optimal ion parameter pair for generating a good-quality nanopore in a G/M heterostructure is 200 eV, 1.27 × 10^16^ /cm^2^, while it is 300 eV, 1.27 × 10^16^ /cm^2^ for an M/G heterostructure. The difference is induced by the different irradiation tolerances of the graphene and MoS_2_ layers. For the case of an M/G heterostructure, and with careful control of the irradiation parameters, it is possible to create a nanopore in the MoS_2_ layer only, while keeping the graphene layer undamaged.
(3) The as-generated nanopore would result in stress concentration around the nanopore in the heterostructure during a stretching process, which leads to the initiation of a crack at a small tensile strain at the nanopore edge. The increase of nanopore size intensifies the stress intensity factor, further reducing the mechanical strength. However, an increase of ion energy and ion dose have limited effect on the mechanical properties of the nanopore structure. By switching the stacking order, the damage sequence of the heterostructure nanopore can be controlled.

## Data Availability

The data that support the findings of this study are available from the corresponding authors upon reasonable request.
